# Generation of a tyrosine hydroxylase-2A-Cre knockin non-human primate model by homology-directed-repair-biased CRISPR genome editing

**DOI:** 10.1016/j.crmeth.2023.100590

**Published:** 2023-09-14

**Authors:** Sho Yoshimatsu, Junko Okahara, Junko Yoshie, Yoko Igarashi, Ryusuke Nakajima, Tsukasa Sanosaka, Emi Qian, Tsukika Sato, Hiroya Kobayashi, Satoru Morimoto, Noriyuki Kishi, Devin M. Pillis, Punam Malik, Toshiaki Noce, Hideyuki Okano

**Affiliations:** 1 Department of Physiology, School of Medicine, Keio University, Shinjuku-ku, Tokyo 160-8582, Japan; 2 Laboratory for Marmoset Neural Architecture, RIKEN Center for Brain Science, Wako City, Saitama 351-0198, Japan; 3 Central Institute for Experimental Animals, Kawasaki City, Kanagawa 210-0821, Japan; 4 Division of Experimental Hematology and Cancer Biology, Cancer and Blood Diseases Institute (CBDI), Cincinnati Children’s Hospital Medical Center (CCHMC), Cincinnati, OH 45229, USA; 5 Division of Hematology, CBDI, CCHMC, Cincinnati, OH 45229, USA; 6 Department of Pediatrics, University of Cincinnati College of Medicine, Cincinnati, OH 45229, USA

## Abstract

Non-human primates (NHPs) are the closest animal model to humans; thus, gene engineering technology in these species holds great promise for the elucidation of higher brain functions and human disease models. Knockin (KI) gene targeting is a versatile approach to modify gene(s) of interest; however, it generally suffers from the low efficiency of homology-directed repair (HDR) in mammalian cells, especially in non-expressed gene loci. In the current study, we generated a tyrosine hydroxylase (*TH*)*-2A-Cre* KI model of the common marmoset monkey (marmoset; *Callithrix jacchus*) using an HDR-biased CRISPR-Cas9 genome editing approach using Cas9-DN1S and RAD51. This model should enable labeling and modification of a specific neuronal lineage using the Cre-*loxP* system. Collectively, the current study paves the way for versatile gene engineering in NHPs, which may be a significant step toward further biomedical and preclinical applications.

## Highlights

• We generate a TH-2A-Cre knockin marmoset model
• Cas9-DN1S and Rad51 proteins facilitate knockin in marmoset zygotes
• Introduced Cre expression is validated through reprogramming
• Introduced Cre activity is validated in an *in vitro* model

## Motivation

Gene engineering in the common marmoset has the potential to provide unique insights into human disease and brain function because of its similarity to humans. To expand the utility of the model system, we attempted and succeeded in generating a gene knockin marmoset using CRISPR-Cas9-mediated and HR-based genome editing. By targeting *TH*, a specific marker for dopaminergic, adrenergic, and noradrenergic neurons, we produced a highly faithful TH reporter system in the marmoset via genome editing.

## Graphical abstract

**Figure:**
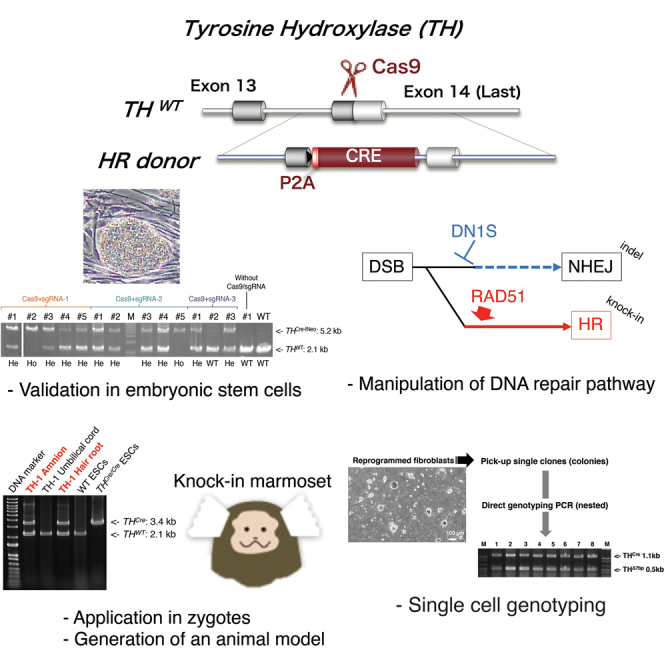

## Teaser

Yoshimatsu et al. generate a TH-2A-Cre knockin marmoset model. Their approaches include validating the knockin construct in embryonic stem cells, injecting Cas9-DN1S and Rad51 proteins with crRNA/tracrRNA to enhance knockin efficiency, genotyping embryos thorough whole-genome amplification, and reprogramming somatic fibroblasts for validation of the knocked-in reporter.

## Introduction

As one of the human-closed NHP models, the marmoset has many advantages for neuroscience research, such as its ease of use, high fecundity, and short periods of gestation and sexual maturation,^1^^,^^2^ compared with macaque monkeys. Using the developed manipulation technology of marmoset early-stage embryos followed by transfer to surrogates, we and other groups have previously succeeded in efficient generation of gene-modified marmosets by lentiviral transgenesis^3^ and gene knockout (KO) using zinc-finger nuclease (ZFN) and Transcription activator-like effector nuclease (TALEN)^4^ for disease modeling and evolutional studies.^4^^,^^5^^,^^6^^,^^7^^,^^8^^,^^9^ In addition, using *Streptococcus pyogenes* CRISPR-Cas9,^10^^,^^11^ we also reported efficient knockin (KI) (at 24%–33% KI efficiency) in marmoset early-stage embryos to introduce point mutation(s) into endogenous gene loci *in vitro*.^12^^,^^13^ However, to the best of our knowledge, the capacity of KI marmoset embryos for full-term development until birth (about 143–147 days in marmosets) remains elusive, and any attempts to introduce reporter gene(s) by KI in marmoset embryos have not yet been reported.

Cre recombinase originally derived from enterobacteria phage P1 is a useful reporter for labeling a specific lineage of cells and conditional control of gene expression.^14^ Cre recognizes a 34-bp *loxP* sequence and causes highly specific recombination, which results in excision or flipping of a *loxP*-flanked sequence in a *loxP* orientation-dependent manner. Genetic reporter systems using the *Cre* gene have been widely used in many model organisms by combinatorial usage of defined promoter sequences or KI. The accuracy of genetic reporters in the former approach (artificial transgene promoter) is affected by multiple epigenetic/topological and currently unknown effects of transgene-integrated loci. Thus, we sought to and achieved to establish a reliable KI-based Cre reporter system in non-human primates (NHPs) *in vivo* for the first time.

## Results

### *In vitro* validation of the *TH-2A-Cre* KI construct using marmoset embryonic stem cells (ESCs)

**Figure 1:**
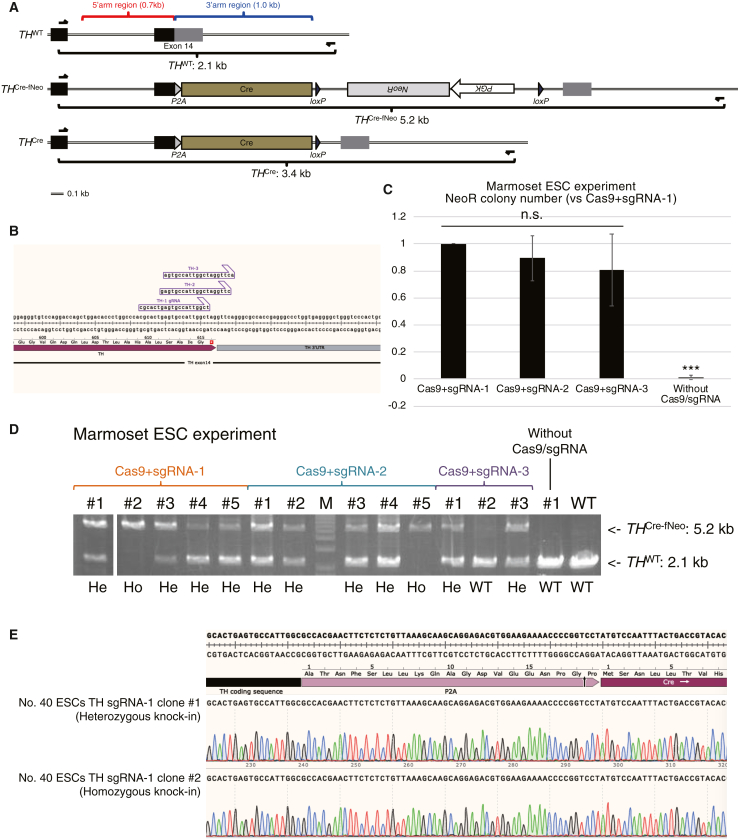
*In vitro* validation of the *TH-2A-Cre* KI construct using marmoset ESCs (A) Schematics of the marmoset *TH*^*WT*^, *TH*^*Cre-fNeo*^, and *TH*^*Cre*^ allele. Homology arm regions in the *TH-2A-Cre(-fNeo)* vector are shown in red (5′) and blue (3′). Primer binding sites for genotyping PCR are shown as black arrows. Endogenous exons, such as the coding region and 3′ UTR, are shown as black and gray boxes, respectively. (B) Three sgRNA sequences (sgRNA-1, -2, and -3) and the PAM sequence (NGG) for the marmoset *TH* gene were used. (C) Relative NeoR colony numbers following G418 selection of marmoset ESCs (n = 3). The colony number of the Cas9+sgRNA-1 condition is set as 1. n.s., not significant. (D) Genotyping PCR of NeoR ESC clones under the following conditions: Cas9+sgRNA-1, -2, and -3 and without Cas9/sgRNA (only the targeting vector was transfected). Ho, homozygous; He, heterozygous. (E) Sanger sequencing of KI alleles (the 5.2-kb bands shown in Figure 1D) of Cas9+sgRNA-1 #1 (He KI) and Cas9+sgRNA-1 #2 (Ho KI) clones. Precise KI was confirmed by sequencing of the junction of the 5′ side of *P2A-Cre* (shown above) and 3′ side (data not shown).

The tyrosine hydroxylase (*TH*) gene is specifically expressed in dopaminergic, noradrenergic, and adrenergic neurons in the central nervous system (CNS). *TH* encodes an important enzyme that catalyzes the conversion of L-tyrosine to l-3,4-dihydroxyphenylalanine (L-DOPA), the precursor of multiple neurotransmitters such as dopamine, norepinephrine (noradrenaline), and epinephrine (adrenaline). Although a previous attempt to label *Th* expression using a KI mouse line harboring the yellow fluorescent protein gene replaced with the *Th* first exon were not fully successful because of the putative importance of the intronic regulatory region(s),^15^ another study using human induced pluripotent stem cells (iPSCs) succeeded in almost complete labeling of *TH* expression by KI of the red fluorescent protein gene into the *TH* termination codon (in the 14th exon) with a self-cleaving *2A* peptide sequence.^16^ Moreover, *Th-IRES-Cre* KI rats (*IRES-Cre* was introduced into the 3′ UTR of the *Th* gene locus) showed *Cre* expression successfully mimicking endogenous *Th* expression despite a slight decrease in *Th* expression from the KI allele.^17^^,^^18^ Given these observations, we initially constructed a *2A-Cre* KI vector targeting the termination codon of the marmoset *TH* gene, located in its 14th exon (Figure 1A, top). We used the *TH-2A-Cre* targeting vector harbored 0.7-kb 5′ homology and 1.0-kb 3′ homology arms with a floxed neomycin resistance cassette (*fNeo*; floxed *PGK-NeoR*) (for ESC experiments; Figure 1A, center) or without (for embryo experiments; Figure 1A, bottom).

Initially, using the *TH-2A-Cre-fNeo* targeting vector, we attempted *in vitro* validation of the *TH-2A-Cre* KI construct. To use CRISPR-Cas9, we designed and tested three single-guide RNAs (sgRNAs) in marmoset ESCs targeting the vicinity of the *TH* termination codon (Figure 1B). We transfected the *TH-2A-Cre-fNeo* vector with or without the CRISPR-Cas9/sgRNA (containing sgRNA1-3) vector into marmoset ESCs. Then, ESCs were selected using G418 (an analog of neomycin), followed by counting the G418-resistant (NeoR) colonies. As a result, the NeoR colony numbers between the three sgRNAs were not significant but significantly improved from the control (without CRISPR-Cas9/sgRNA) (Figure 1C). Moreover, by genotyping PCR, we confirmed that most of the NeoR ESC clones in the ESC experiment were heterozygous (He) or homozygous (Ho) KI (Figure 1D), as expected of previous observations that we hardly obtained wild-type (WT) clones following drug selection of marmoset ESCs in KI experiments.^12^^,^^19^ The precise *2A-Cre* introduction by KI was confirmed using Sanger sequencing (Figure 1E). We chose sgRNA-1 for further experiments using early-stage embryos because of its slightly better performance in NeoR colony formation among the three sgRNAs (Figure 1C).

### Evaluation of KI efficiency in marmoset early-stage embryos and production of a KI marmoset model

**Figure 2:**
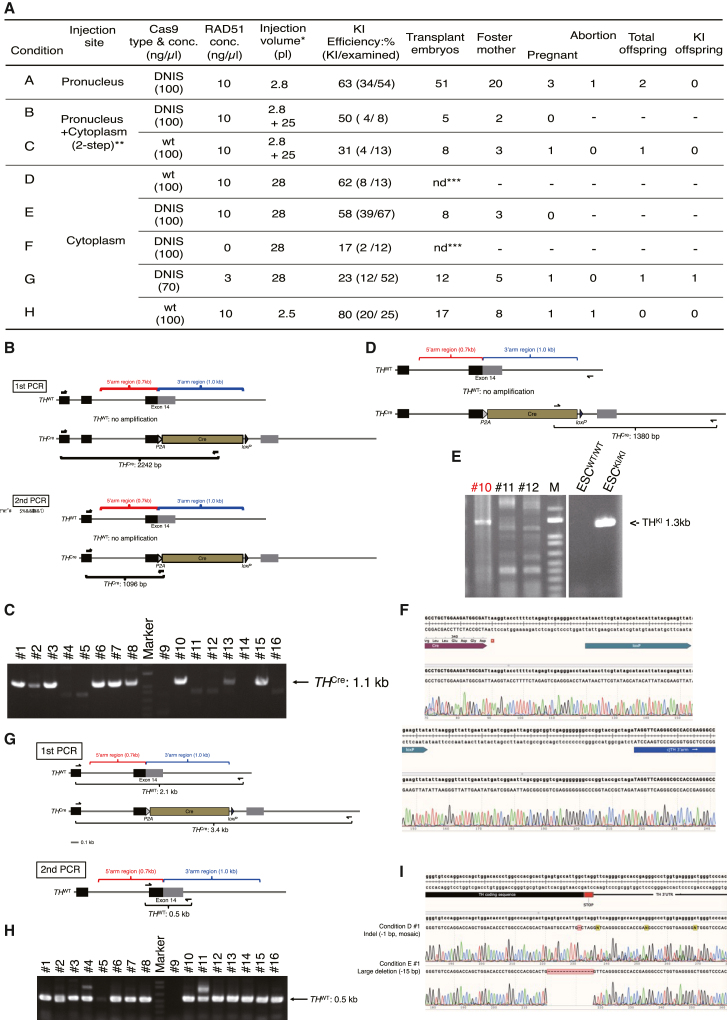
Evaluation of genome editing efficiency in marmoset early-stage embryos (A) A summary table of early-stage embryo experiments and KI efficiencies under eight conditions (A–H). ∗, the volume was calculated by the size of the injected droplet diameter. ∗∗, in the 2-step injection, donor DNA (100 ng/μL) was injected into the pronucleus, and then RAD51, Cas9, and crRNA+ tracrRNA (50 ng/μL) were injected into the cytoplasm. ∗∗∗, not done. ca., circa. (B) A schematic of the 2-step PCR analysis for KI allele detection (5′ side). In both PCRs, we used 5′-external primers to not amplify the KI vector itself. (C) A representative image of the result of genotyping PCR analysis using amplified genomic DNA (gDNA) from marmoset early-stage embryos. *TH-2A-Cre* KI was detected by the KI-specific 1.1-kb DNA bands. In this experiment (condition E), 9 of 16 embryos were considered KI positive. (D) A schematic of 1-step PCR analysis for KI allele detection (3′ side). We used 5′-internal and 3′-external primers to amplify only the KI allele. (E) A representative image of the result of genotyping PCR analysis (3′ side) using amplified gDNA from marmoset early-stage embryos. In this experiment (condition E; same samples as used in C), #10 was considered knocked in. (F) DNA sequencing analysis of the KI allele of #10 (3′ side). (G) A schematic of the 2-step PCR analysis for WT allele detection. In the first PCR, we used 5′- and 3′-external primers to not amplify the KI vector and KI allele. (H) A representative image of the result of genotyping PCR (WT allele) analysis using amplified gDNA from marmoset early-stage embryos. In this experiment (condition E; same samples as used in C), all embryos were considered to harbor *TH*^WT^ allele(s). (I) Representative images of DNA sequencing analysis of the *TH*^WT^ allele. As described in the main text, ∼20% (Cas9-DN1S) and ∼30% (Cas9-WT) of embryos harbored indels or large deletion allele(s). Details of each condition are shown in (A).

Next, we evaluated the efficiency of TH-2A-Cre KI in early-stage marmoset embryos. Based on a previous method,^12^ we performed microinjection of purified Cas9 protein, an annealed CRISPR RNA (crRNA) (TH sgRNA-1 sequence) with a *trans* CRISPR RNA (tracrRNA), DNA donor vector (the *TH-2A-Cre* vector without *fNeo*) into two pronuclear (2PN)-stage marmoset embryos following *in vitro* fertilization. Moreover, we tested two candidates for enhancing KI efficiency as follows: (1) usage of purified Cas9-DN1S protein,^20^ a chimeric Cas9 fused to a dominant negative mutant of human P53BP1, instead of WT Cas9, and (2) supplementation with RAD51, a critical factor for homology-directed repair in mammalian embryos,^21^ in the microinjection solution. We testified eight conditions (Figure 2A).

**Figure 3:**
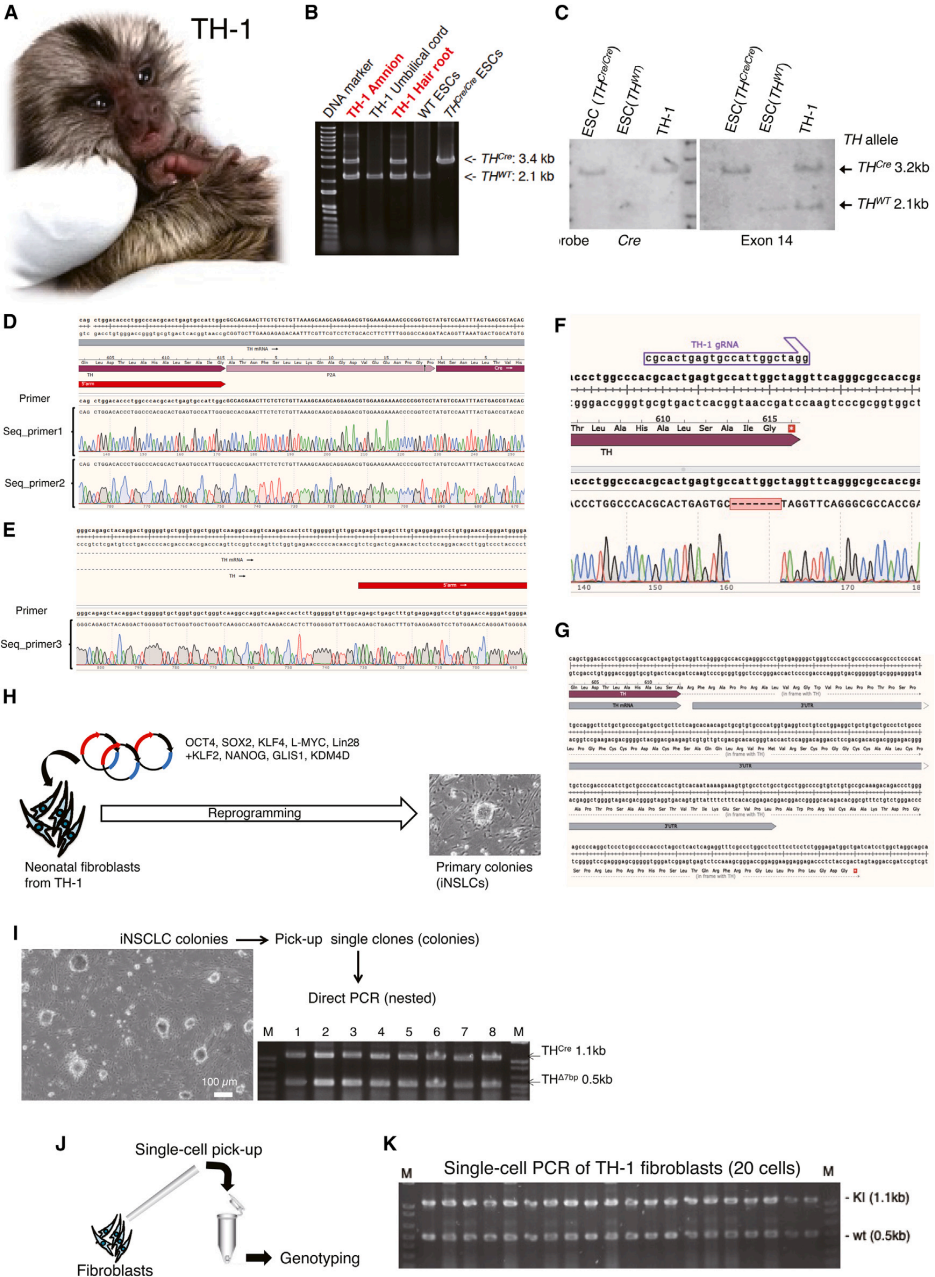
Characterization of the TH-2A-Cre marmoset model (A) A representative macroscopic image of TH-1 after birth. (B) Genotyping PCR analysis of TH-1 tissues and positive/negative control ESCs. The same primers as shown in Figure 1D were used. (C) Southern blotting analysis using probes specific for *TH* exon 14 (left) and *Cre* (right) sequences. We used 10-μg genomic DNA for the electrophoresis. (D–F) DNA sequencing of TH-1 in the KI allele (D and E) and the putative WT allele with a 7-bp deletion (F) in the *TH* gene locus. (G) The 7-bp deletion in the non-KI *TH*^*WT*^ allele rendered the coding sequence longer, which may add superfluous amino acid residues (from 614Arg; ∼115 aa, ∼12.1 kDa) in the C terminus. (H) Reprogramming experiments. Fibroblast images (left) were cropped from our previous study.^22^ We show a representative phase-contrast image of primary colonies (iNSLCs) derived from TH-1 fibroblasts by reprogramming (right). (I) Genotyping PCR results showed that all eight iNSLC colonies (clones) were He for KI. (J and K) Single-cell PCR was performed using 20 TH-1 dermal fibroblasts. M, DNA marker.

Following microinjection into the pronucleus and/or cytoplasm of marmoset 2PN embryos (day 0), we sampled day 3 embryos (at the 4- to 12-cell stage) for genotyping PCR analysis following whole-genome amplification (WGA). To enhance the detection efficiency of the KI allele, genotyping PCR for WGA samples was performed by an optimized 2-step nested PCR method (Figure 2B, top and center). KI embryos were identified by the 1.1-kb KI-specific PCR band (Figure 2B, bottom), and precise KI was confirmed (Figure 2C). We also confirmed precise KI by 1-step PCR of the 3′ side (Figures 3D and 3E). Moreover, we investigated the insertion or deletion (indel) and large deletion efficiency. We revealed that ∼20% and ∼30% of embryos harbored indels or large deletion allele(s) by microinjection of Cas9-DN1S and WT, respectively (Figures 2F and 2G). We note that most KI embryos (∼99%) were He or mosaic (Figure 2F); therefore, it is unlikely that possible embryonic lethality of Ho KI mutants affects the full-term developmental potential of transplanted embryos. In addition, we faced the difficulty in detecting the long PCR amplicons (over 1.5 kb) from early-stage embryo WGA samples (it was difficult to detect KI by using 5′ and 3′ external primers simultaneously).

As summarized in Figure 2A, various KI efficiencies were obtained under respective conditions (A–H). Although we confirmed the RAD51-mediated enhancement of KI efficiency (KI: 17% under 0 ng/μL RAD51 condition F compared with KI: 58% under the similar condition E with 10 ng/μL RAD51), we could not obtain any pregnancies under condition E. Moreover, we performed additional approaches (conditions A–C), such as microinjection into the pronucleus or pronucleus + cytoplasm (by a 2-step method^13^). Under these conditions, we observed an increase in KI efficiency with Cas9-DN1S (Figure 2A). However, although we obtained three offspring under these conditions in total, the resultant three offspring were negative for any genome edits in the targeted locus, such as KI and indels (data not shown). Accordingly, we inferred that the toxicity of the microinjection solution might restrict the full-term developmental potential of the genome-edited embryos.

To overcome this, we decreased the concentration of components in the injected solution. Under condition G, using 70 ng/μL Cas9-DN1S and 3 ng/μL RAD51, although the KI efficiency was decreased to 23%, we finally obtained one KI offspring (named TH-1, male) harboring a He *TH-2A-Cre* KI allele detected by genotyping PCR without any macroscopic abnormalities (Figures 3A and 3B). Also, we discovered that the decrease (28 pL–2.5 pL) in the quantity of the injected solution with WT Cas9 and RAD51 dramatically improved the KI efficiency (conditions D and H; Figure 2A); however, the low pregnancy rate was not improved, and we could not obtain live offspring. Consistent with our result, Yao et al.^23^ reported that a decrease in microinjection solution improved KI efficiency in macaque monkey embryos. In our study on generation of *MECP2*-KO marmosets by CRISPR-Cas9-mediated DNA cleavage (N.K., unpublished data), we obtained 11 live Ho /hemizygous *MECP2*-KO fetuses from 22 pregnant surrogates using 144 transferred embryos (microinjected) transferred to 56 surrogates (39.3% pregnancy rate). Therefore, we conclude that the low pregnancy rate in *TH-2A-Cre* KI experiments (12.8% in total) may be due to currently unknown factor(s); e.g., the locus effect or toxicity of exogenous DNA itself or a functional *Cre* transcript that causes implantation failure of embryos under microinjection conditions for KI. Furthermore, considering no KI marmoset production from condition A–C even with the three live-birth fetuses (Figure 2A), KI embryos themselves may harbor a restricted potential for full-term development until birth compared with unedited ones.

### Genomic analyses of the *TH-2A-Cre* KI marmoset model

To further confirm KI of TH-1, we performed Southern blotting, which revealed targeted introduction of *2A-Cre* into *TH* exon 14 and that the targeting vector was not integrated into any other loci of the TH-1 genome (Figure 3C). By Sanger sequencing of the KI allele in TH-1, we confirmed precise (in-frame) introduction of *2A-Cre* (Figure 3D) and that the junction of the 5′ homology arm and external region was intact (Figure 3E). Moreover, whole-genome sequencing (WGS) analysis revealed no evident off-targets in the TH-1 genome compared with the reference (Figure S1) and relatives (Figure S2). Furthermore, by Sanger sequencing of the putative WT allele in TH-1, we noticed that there was a 7-bp deletion in the vicinity of the cleavage site by Cas9 and sgRNA (Figure 3F), which added superfluous amino acid residues in the C terminus of the *TH* gene (Figure 3G).

To explore the possibility of mosaicism in TH-1 somatic cells, we reprogrammed skin-derived fibroblasts of TH-1. As described previously,^22^ we reprogrammed fibroblasts into colony-forming cells, such as induced neural stem cell-like cells (iNSLCs) by transfection of nine reprogramming factors (*OCT4*, *SOX2*, *KLF4*, *LMYC*, *mp53DD*, *KLF2*, *NANOG*, *GLIS1*, and *KDM4D*) using an episomal vector system and induction medium naive induction medium (NSM) (Figure 3H). Following derivation of primary colonies, we mechanically isolated eight colonies (consisting of iNSLCs) and genotyped them. As a result, we detected the same pattern of PCR bands, which showed He *2A-Cre* KI with a 7-bp-deleted non-KI allele (Figure 3I). Because of the principle of reprogramming (a single fibroblast cell is reprogrammed to form a clonal colony), we concluded that TH-1 is He for *TH*^*Cre*^ without any off-targets except a 7-bp deletion in the non-KI TH allele (Figures 1 and 2). Also, we performed single-cell PCR using 20 TH-1-derived dermal fibroblasts and confirmed that TH-1 is He for *TH*^Cre^ (Figures 3J and 3K).

### *In vitro* functional analysis of the TH-Cre KI allele and point mutation of Cre

**Figure 4:**
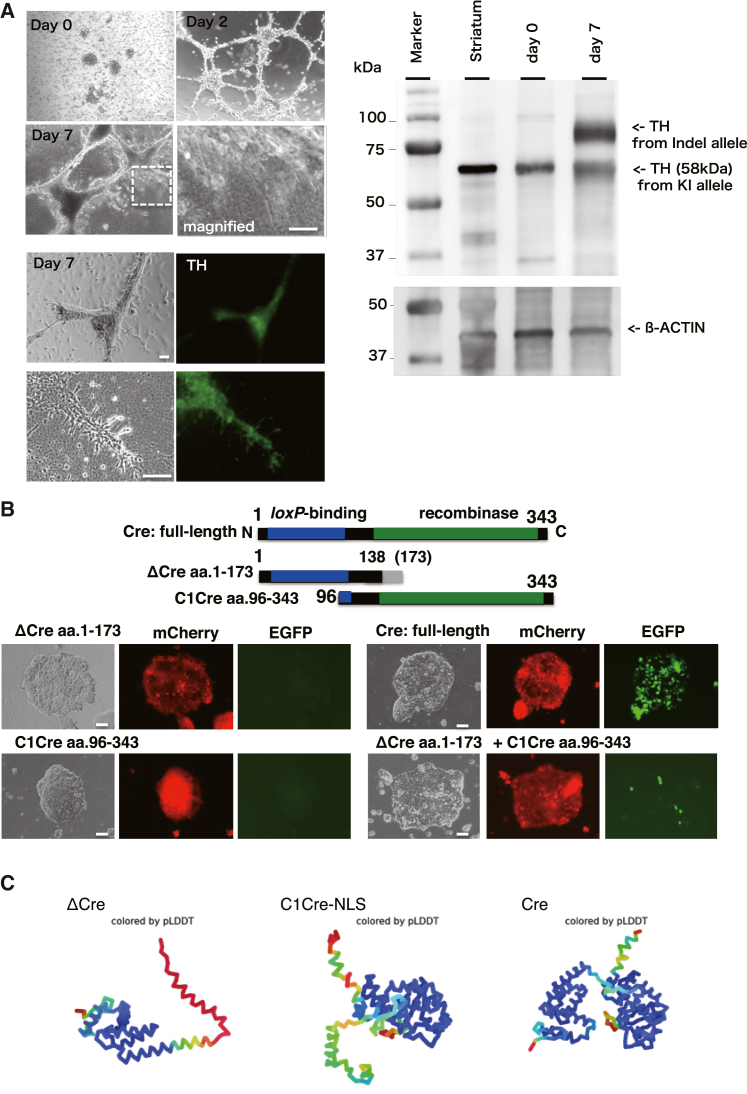
Directed differentiation of TH-1 iNSLCs and *in vitro* validation of Cre recombination (A) Left: representative phase-contrast/green fluorescence images of differentiated iNSLCs derived from TH-1 fibroblasts (Figure 3) on days 0, 2, and 7. For immunocytochemistry of TH, we used a TH-specific primary antibody and an Alexa 488-conjugated secondary antibody. Scale bars, 100 μm. Right: western blotting of TH and β-actin (internal control). Extracted protein solution (approximately 10 μg/lane) from adult marmoset striatum differentiated TH-1 iNSLCs on days 0 and 7 was applied and immunodetected as described previously described.^24^. (B) Split-Cre approach. Top: schematics of full-length Cre (1–343 aa), TH-1 mutated Cre (ΔCre: 1–138 aa was intact, total 173 aa), and C1Cre (96-343aa.).^25^ The experimental procedures for the split-Cre experiment are described in the Supplemental experimental procedures. Scale bar, 100 μm. (C) AlphaFold2-mediated^26^^,^^27^ structural estimation of Cre variants in this study.

To confirm the functionality of *2A-Cre* for *loxP*-specific recombination *in vitro*, we used iNSLCs established from TH-1 fibroblasts as described above (Figures 3H and 3I). By directed neural differentiation of TH-1 iNSLCs to the dopaminergic neuronal lineage, we confirmed TH-positive neuron-like cells with neurite-like structures (Figure 4A, left). We confirmed TH expression in differentiated cells by western blotting (Figure 4A, right). We note that the TH protein encoded from the non-KI *TH* allele with a 7-bp deletion (Figure 3F) showed an immunoreactive band over 75 kDa, which is consistent with the extra peptides caused by the presumed C terminus elongation (Figure 3G).

In addition, during these experiments, we found a frameshift mutation in *Cre* (c.419delC; p.T140Mfs∗173) of TH-1 in the KI allele, which may result from the toxicity of *Cre* per se in primate development. Because this frameshift impairs Cre function for *loxP*-specific recombination, we tested the split-Cre system,^25^ which was devised to avoid the cellular toxicity of full-length Cre by its long-term expression (Figure 4B, top). Because TH-1 *Cre* (named *ΔCre*) encodes an intact amino acid (aa) sequence from 1–139 aa (full length, 343 aa), we chose *C1Cre* consisting of *Cre*’s 96–343 aa^25^ (Figure 4B, center). HEK293T cells carrying a color-switch reporter (CAG promoter-driven *mCherry* was switched to *EGFP* by Cre-mediated *loxP* recombination) were used for the *in vitro* validation experiment. By transfecting *ΔCre* and *C1Cre*, although single transfection of *ΔCre* did not induce Cre recombination (Figure 4B, bottom left), we found that 18.8% ± 1.25% EGFP fluorescence (n = 6) resulted from Cre recombination by the Split-Cre approach (co-transfection of *ΔCre* and *C1Cre*) compared with transfection of full-length *Cre* (Figure 4B, bottom right).

## Discussion

In summary, the current study represents the first generation of an NHP model harboring a KI reporter gene for specific neural linages, such as dopaminergic, adrenergic, and noradrenergic ones in the *TH-2A-Cre* model, which is distinct from two previous reports on generation of KI macaque monkeys with *ACTB-P2A-mCherry*^23^ and *OCT4-hrGFP*^28^ targeting highly expressed loci in early-stage embryos. Moreover, to the best of our knowledge, this study reports the first generation of a KI model in the marmoset, which has not been achieved in previous attempts^12^^,^^13^ because of the difficulty of the full-term development of KI embryos. The results in the present study expand the scope of biomedical and preclinical research using the marmoset model because the reported gene engineering approaches in the model relied on lentivirus-mediated transgenesis^3^^,^^5^^,^^7^^,^^8^^,^^29^^,^^30^^,^^31^ and ZFN/TALEN/Cas9-mediated gene KO.^4^^,^^9^^,^^32^

Although further optimization for robust production of KI marmosets is still required, especially exploring the biological factor(s) that prevent(s) full-term development and successful implantation of KI embryos, is beneficial, addition of KI technology to production of the marmoset model can be applied to introduce pathogenic or evolutionary mutation(s), reporter gene(s) in a specific gene locus, which is critically important for development of faithful disease modeling or evolutionary analysis in further studies. For example, introduction of pathogenic mutation(s), including the A30P mutation in alpha-synuclein (*SNCA*)^33^^,^^34^ and NL-G-F/NL-F mutations in amyloid precursor protein (*APP*),^35^ is useful for disease modeling of Parkinson’s and Alzheimer’s diseases with high penetrance, respectively. Furthermore, KI technology can make it feasible to replace an entire endogenous gene locus with one of another species, including humanization of an evolution-related gene.

The utility of the *TH-2A-Cre* KI line described in the present study would be eclectic, including TH-positive neuron-specific Ca imaging (using *GCamP*^36^) and manipulation (using designer receptors exclusively activated by designer drugs [DREDDs]^37^), and viral tracing for neural circuit analysis.^38^ Because of the difficulty of precise KI in NHPs, the present study paves the way for novel approaches for primate-specific neurological and pathological analyses in future studies.

Moreover, the TH-1 marmoset harbors a 7-bp deletion in the non-KI allele, which adds over 10 kDa extra peptides in the C terminus (Figure 3G). The TH enzyme functions as a tetramer, and the C terminus is reportedly the tetramerization domain,^39^ therefore this mutation may impair the enzymatic function of TH. Consistently, the TH-1 marmoset showed TH-deficient-like phenotypes such as expressionless and tremors in the limbs from 5-month-old. These symptoms were ameliorated by L-DOPA administration (Figure S3; Videos S1, S2, and S3). Thus, if this putative dominant-negative effect can also be confirmed in the next generation by *in vitro* fertilization with WT oocytes, then the strain provides us with a primate TH-deficient model, which is useful for drug discovery and development of novel therapeutic approaches.

Video S1. TH-1 pre-DOPA (5 months) scene 1, related to Figure 3

Video S2. TH-1 pre-DOPA (5 months) scene 2, related to Figure 3

Video S3. TH-1 post-DOPA (70 days of administration), related to Figure 3

The split-Cre approach^25^ has made the frameshift-included *Cre* (*ΔCre*) functional, but the comparatively low efficiency of *loxP*-specific recombination has room for improvement. The continuous generation approach may make it possible to generate a TH-2A Cre marmoset model carrying functional full-length *Cre*. In addition, structural estimation^26^ of *ΔCre* (Figure 4C) for acquisition of the specific recombination function with an optimized C-terminal *Cre* sequence may improve the utility of the current *TH-2A-Cre* model. Moreover, although our preliminary approach of prime editing using spCas9n-based PE2^40^ for repairing *ΔCre* was currently inefficient (Figure S4), which was restricted by the targeting scope of the spCas9 protospacer adjacent motif (PAM) sequence in the vicinity of the mutation in *ΔCre*. More improved methods of prime editing or recombination may enhance the editing (repair) efficiency. This may be a limitation of the present study; we still need to explore an efficient method to make *ΔCre* fully functional as a recombination reporter *in vivo*. Considering that we could not obtain a live-born monkey with fully functional *Cre* through intensive efforts, the toxicity of full-length Cre protein in primate development to term may be the limitation. Further studies are needed to clarify this issue.

Multimodal similarities especially in the CNS between NHPs and humans, render NHPs experimentally advantageous for neuroscience and preclinical studies. Thus, the development of KI technology in NHPs may help enhance our knowledge of higher brain functions, diseases, and primate-specific evolutional features. More recently, adenovirus-associated virus (AAV) vectors with the primate blood-brain barrier-penetrating potential for whole-brain transfection have been devised.^41^^,^^42^ However, the stringent size limitation of AAV transgenes in the physical principle for packaging (∼4.8 kb transgene between inverted terminal repeats) restricts the appropriate choice of a transgene-driving promoter. Therefore, KI technology of production in NHPs may facilitate specific targeting of cell type(s) with the combination of the newly devised AAV technology for analyses of neural circuits, neuropathology, and evolutional acquisition of human/primate-specific neural traits.^5^^,^^43^

Furthermore, because marmoset monkeys have higher reproductive efficiency and shorter gestation/sexual maturation periods than macaque monkeys,^1^^,^^2^ the first generation of KI marmosets described in the current study holds great promise for further application of KI model animals for a robust platform for recapitulating human pathology and devising novel therapeutic innervations.

### Limitation of the study

While we succeeded in generation of a *TH-Cre* KI marmoset model, there are still limitations. First, the integrated *Cre* gene was mutated; therefore, to use the reporter system, we need to repair or re-functionalize the mutated *Cre*. In our approaches, including split-Cre and prime editing, we demonstrated Cre-mediated recombination but with comparatively low efficiency. Thus, it is still required to enhance the efficiency of Cre-mediated recombination. Second, compared with our Cas9-mediated KO approach, successful birth of a KI marmoset was insufficient. Scrutinizing the possible toxicity of any used reagents in this study will be beneficial for further generation of KI marmoset models. Third, although we already obtained offspring of TH-1 (unpublished data), increasing the number of the KI animals is important for neuroscience research. Efficient methods for reproduction should be devised for robust usage of gene-engineered marmosets.

## STAR★Methods

### Key resources table

**Table:** 

REAGENT or RESOURCE	SOURCE	IDENTIFIER
**Antibodies**		

Rabbit anti-TH antibody	Abcam	Cat#ab76442; RRID: AB_1524535
Alexa Fluor 488-conjugated anti-rabbit IgG	Abcam	Cat#ab150077; RRID: AB_2630356
Mouse anti-β-actin antibody	Sigma	Cat#MABT219; AB_11203498

**Bacterial and virus strains**		

One Shot™ Stbl3™ Chemically Competent E. coli	Thermo Fisher Scientific	Cat#C737303

**Biological samples**		

Marmoset oocytes	This paper	N/A
Marmoset sperms	This paper	N/A
Marmoset fibroblasts	This paper	N/A
Marmoset iNSLCs	This paper	N/A

**Chemicals, peptides, and recombinant proteins**		

Alt-R™ S.p. Cas9 Nuclease V3	IDT	Cat#1081058
Cas9-DN1S	Jayavaradhan et al.^20^	N/A
Custom Alt-R™ CRISPR-Cas9 guide RNA	IDT	N/A
Alt-R® CRISPR-Cas9 tracrRNA	IDT	Cat#1072532
(RAD51-134H) Recombinant Human RAD51	Creative Biomart	Cat#RAD51-134H

**Critical commercial assays**		

REPLI-g Single Cell Kit	Qiagen	Cat#150343
PrimeSTAR® Max DNA Polymerase	Takara	Cat#R045A
BigDye Terminator v1.1 cycle sequencing kit	Thermo Fisher Scientific	Cat#4337449

**Deposited data**		

Raw data of WGS	This paper	DRA013552 and DRA016136

**Experimental models: Cell lines**		

CMES40 (No.40)	Sasaki et al.^44^	N/A
HEK293T	Provided by Dr. Hiroyuki Miyoshi at Keio University	N/A
TH-1 fibroblasts	This paper	N/A
Th-1 iNSLCs	This paper	N/A

**Experimental models: Organisms/strains**		

*Marmosets (Callithrix jacchus)*	RIKEN in-house colony	N/A

**Oligonucleotides**		

TH sgRNA-1: CGCACTGAGTGCCATTGGCT	This paper	N/A
TH sgRNA-2: GAGTGCCATTGGCTAGGTTC	This paper	N/A
TH sgRNA-3: AGTGCCATTGGCTAGGTTCA	This paper	N/A

**Recombinant DNA**		

pSpCas9(BB)-2A-Puro (PX459)	Addgene	Cat#62988
pKI-cjTH-Cre-rfNeo	Addgene (deposited)	Cat#186248
pKI-cjTH-Cre-Δlox	Addgene (deposited)	Cat#186249
pCE-hOCT3/4	Addgene	Cat#41813
pCE-hSK	Addgene	Cat#41814
pCE-hUL	Addgene	Cat#41855
pCE-mp53DD	Addgene	Cat#41856
pCXWB-EBNA1	Addgene	Cat#36724
pCE-K2N	Addgene	Cat#154879
pCE-KdGl	Addgene	Cat#154880
CAG-Cre	This paper	N/A
CAG-TH-2A-ΔCre	This paper	N/A
U6-pegRNA	Addgene	Cat#132777
CMV-PE2	Addgene	Cat#132775

**Software and algorithms**		

Integrative Genomic Viewer	Robinson et al.^45^	https://software.broadinstitute.org/software/igv/

**Other**		

Marmoset genome reference	Washington University	WUGSC 3.2/calJac3

### Resource availability

#### Lead contact

Further information and requests for resources and reagents shold be directed to and will be fulfilled by the lead contact, Hideyuki Okano (hidokano@keio.jp).

#### Materials availability

This study did not generate new unique reagents.

### Experimental model and study participant details

#### Marmosets

The marmosets used in the current study were 2–8 years old (average weight from 330 to 550 g). The marmosets were pair/family-housed under a warm and humid condition (27 ± 2°C, 55 ± 20% humidity). In total, 83 female marmosets were used as oocyte donors and 35 male marmosets were used as sperm donors. Marmosets were obtained from an in-house breeding colony at RIKEN Center for Brain Science.

All protocols for animal experiments were performed in accordance with the guidelines for laboratory animals set forth by the National Institutes of Health, and the Ministry of Education, Culture, Sports, Science and Technology (MEXT) of Japan, and were approved by the Institutional Animal Care and Use Committee of the RIKEN (approval No. W2021-2-037(2) and W2021-2-037(4)). Animal care was conducted in accordance with the National Research Council (NRC) Guide for the Care and Use of Laboratory Animals (2011).

#### Cell lines

A female common marmoset ESC line, No. 40 (CMES40^44^) was used in the current study. ESCs were cultured as described previously.^12^ HEK293T cells (kindly provided by Dr. Hiroyuki Miyoshi at Keio University) were cultured as described previously.^46^

### Method details

#### Preparation and microinjection of 2PN embryos, and embryo transfer to surrogates

The foster mothers were kept pairwise with vasoligated males. Vasoligation, oocyte and sperm collection, and *in vitro* fertilization were performed as previously described.^4^ Preparation of microinjection solution, and microinjection into 2PN marmoset embryos was performed as previously described^12^ with slight modifications. In brief, we used purified recombinant proteins as follows: WT Cas9 (IDT), Cas9-DN1S^20^ and human RAD51 (Creative Biolabs) at the concentrations described in Figure 2A. As the crRNA, we used sgRNA-1 for marmoset *TH* gene (CGCACUGAGTGCCAUUGGCU). The crRNA and tracrRNA were purchased from IDT. The crRNA and tracrRNA were annealed (50 ng/μL) and incubated with the Cas9 protein to form the RNP complex. The RNP complex, DNA vector (100 ng/μL) and RAD51 protein were diluted in nuclease-free water (Qiagen). For the 2-step injection method, the microinjection solution containing the DNA vector was prepared separately. Microinjection was performed using a FemtoJet 4i device (Eppendorf). The volume of injected fluid is regulated by the inner diameter of the needle, the injection pressure, and the injection time. We performed injections into zygotes under different conditions (as shown in Figure 2A) and measured the diameter of the spheres identified by the interface of the injected solution. The injection volume is an estimate calculated from the diameter, assuming that the same volume was injected using the same liquid components and injection conditions were used.

#### DNA vectors

As Cas9-gRNA vectors, we used PX459^11^-based vectors in which sgRNA1-3 sequences (shown in Figure 1B) were subcloned respectively. The subcloned sgRNA sequence for PX459 are as follows: sgRNA-1 (CGCACTGAGTGCCATTGGCT), sgRNA-2 (GAGTGCCATTGGCTAGGTTC), and sgRNA-3 (AGTGCCATTGGCTAGGTTCA). *TH-2A-Cre-fNeo* targeting vector was constructed by inserting synthesized 0.7-kb 5′homology and 1.0-kb 3′homology arm fragments with *fNeo* into pCR-BluntII-TOPO (Thermo Fisher Scientific) for ESC experiments (named pKI-cjTH-Cre-rfNeo, Addgene #186248). For microinjection experiments, the *fNeo* cassette was excised using recombinant Cre protein (New England Biolabs) and named pKI-cjTH-Cre-Δlox (Addgene #186249). DNA vectors used in the present study are available from Addgene (https://www.addgene.org/) or the corresponding authors upon request.

#### Cell transfection and genotyping PCR

Cas9-gRNA and *TH-2A-Cre-fNeo* targeting vector (TV) were prepared at a concentration of 1 μg/μL in Tris-HCl-EDTA buffer (pH 8.0). For transfection, 10 μg of DNA was transfected, which consisted of 8-μg of TV and 2-μg of each Cas9-gRNA vector. For transfection, DNA vectors (total 10 μg), lipofectamine-LTX PLUS reagent (2.5 μL; Thermo Fisher Scientific) and LTX reagent (25 μL; Thermo Fisher Scientific) were added to 500 μL OPTI-MEM (Thermo Fisher Scientific) and added to sub-confluent ESCs cultured in one well of a 6-well plate. Twenty-four hours after transfection, then the cells were dissociated into single-cells using 2.5g/L-Trypsin Solution (trypsin; Nacalai Tesque), centrifuged and trypsin was aspirated, then the cells were suspended in ESM containing Y-27632 (10 μM; Merck Millipore), and re-seeded onto new feeder cells resistant to G418 (day 1). On day 3, the medium was changed to ESM containing G418 (50 μg/mL; Thermo Fisher Scientific). After 2 weeks, the drug-resistant colonies were counted and picked for further cloning.

For genotyping PCR, cells were lysed overnight at 55°C in cell lysis buffer consisting of Tris-HCl (0.2 M), EDTA (10 mM), SDS (0.2%) and NaCl (0.2 M) in nuclease-free water with proteinase K (10 μg/mL). Genomic DNA (gDNA) was purified using a standard phenol-chloroform and ethanol method. PrimeSTAR Max DNA polymerase (Takara Bio) was used for genotyping PCR, according to the manufacturer’s instructions. Purified DNA was stored in Tris-HCl/EDTA buffer (pH 8.0) at a concentration of 50–200 ng/μL. Genotyping PCR was performed as follows: 30 s at 95°C; 35 cycles of 10 s at 98°C and 8 min at 68°C; then 10 min at 68°C; and a final incubation at 4°C until gel electrophoresis. The primers used are as follows: GCCCTACCAAGACCAGACATACC and CTCACAGCCCTTCAGAGACACTC (Amplicon size: WT, 2187 bp; KI, 3515 bp).

For WGA of embryo-derived gDNA (for day-3 embryos (at the 4 to 12-cell stage) following microinjection), we used REPLI-g Single Cell Kit (Qiagen) according to the manufacturer’s instructions. The resultant amplified gDNA was diluted in water at 1:100. Genotyping PCR was performed as follows: 30 s at 95°C; x cycles of 10 s at 98°C and y min at 68°C (x and yvalues are described below); then 10 min at 68°C; and a final incubation at 4°C until gel electrophoresis. For detection of *TH*^*WT*^ and *TH*^*Cre*^ alleles with high sensitivity, we performed 2-step nested PCR. For initial PCR, we used GCCCTACCAAGACCAGACATACC and CTCACAGCCCTTCAGAGACACTC for *TH*^*WT*^ (x = 50, y = 10), or GTACTGGTTCACGGTGGAGTTTG and CCCGGCAAAACAGGTAGTTATTC for *TH*^*Cre*^ (x = 50, y = 4). For secondary PCR, following dilution of the initial PCR solution in water at 1:100, we used AGACTCTGTCCGCTGATTGACC and GAAACCTCTGAGTGAGGCTAGGG for *TH*^*WT*^ (x = 35, y = 1) GCCCTACCAAGACCAGACATACC and CGTCTCCTGCTTGCTTTAACAGA for *TH*^*Cre*^ (x = 35, y = 3).

#### Reprogramming and neuronal differentiation of iNSLCs

Reprogramming of the TH-1 skin-derived fibroblasts was performed as described previously.^22^ Primary colonies of iNSLCs, were mechanically isolated (picked up) for direct PCR. iNSLC culture was performed as described previously^22^ with modifications, including supplementation of 2 μM Thiazovivin (Abcam) and 20 ng/ml bFGF (Reprocell) into the NSM medium.

For differentiation, iNSLCs were transferred to Matrigel-coated (Matrigel MatrixHC, 354262, Corning) chamber slides (Lab-TekII 8-well, Nunc) in Neuralbasal medium (Gibco). Morphological changes were observed from day 2, and neurite-like cilia were evident on day 7. For immunocytochemistry of TH, anti-TH primary antibody (1:400, Abcam, ab76442) and Alexa Fluor 488-conjugated anti-rabbit IgG (1:500, Abcam, ab150077) were used, followed by optical imaging using a BZ-X800 (Keyence).

#### Southern blotting

Southern blotting was performed as described previously.^12^ Genomic DNA was purified as described above and digested with *Pst*I (Takara) overnight followed by phenol and ethanol-based standard DNA purification. For producing digoxigenin (DIG)-labelled probes, we used AGACTCTGTCCGCTGATTGACC and GAAACCTCTGAGTGAGGCTAGGG specific for the marmoset *TH* exon14 (amplicon size: 519 bp, the estimated band sizes are WT: 2.1 kb, KI: 3.2 kb), and GAACCTGATGGACATGTTCA and CCCGGCAAAACAGGTAGTTATTC specific for the *Cre* gene (amplicon size: 650 bp, the estimated band size is KI: 3.2 kb).

#### Gel extraction and DNA sequencing

For DNA sequencing, gel extraction of specific DNA bands was performed as described previously.^12^

DNA sequencing analysis was performed using the BigDye Terminator v1.1 cycle sequencing kit (Thermo Fisher Scientific) with the 3130xl Genetic Analyzer (Applied Biosystems). The sequence data presented in the figures were illustrated using the Snap Gene software (GSL Biotech). As sequencing primers, we used GTACTGGTTCACGGTGGAGTTTG (Seq_primer1), GCCCTACCAAGACCAGACATACC (Seq_primer2), and CGTCTCCTGCTTGCTTTAACAGA (Seq_primer3).

#### WGS and off-target analysis

WGS reads were obtained using the NGS library of genomic DNA of TH-1 derived from its amnion (performed by Azanta Life Sciences, Japan). Mapping of the raw reads on the marmoset genome reference (WUGSC 3.2/calJac3) was performed by STAR as described previously.^47^ Mapped reads were imaged using the Integrative Genomic Viewer^45^ (https://software.broadinstitute.org/software/igv/). Raw data were deposited in DDBJ (https://ddbj.nig.ac.jp/public/ddbj_database/dra/fastq/). DRA Submission: DRA013552 and DRA016136).

#### DNA electrophoresis

DNA electrophoresis was performed using 1% or 2% Agar-gel, 100 V, 30 min. DNA in resultant gels were stained with EtBr (Nacalai Tesque) and imaged by UV. We used 1kb plus DNA ladder (Thermo Fisher Scientific) as a DNA marker.

#### Split-Cre experiments

Lipofectamine LTX was used for plasmid transfection to HEK293T cells according to the manufacturer’s introductions. A lentiviral vector encoding CAG promoter-driven floxed *mCherry* followed by *EGFP* (kindly provided by Drs. Kent Imaizumi and Takefumi Sone at Keio University) was used and transfected to HEK293T cells to establish the color-switch HEK293T cell line which was used in the Split-Cre experiments (Figure 4B) and prime editing approach (Figure S4).

### Quantification and statistical analysis

All data are expressed as mean ± s.e.m. Differences between means were compared using Student’s *t* test. Differences were considered statistically significant at p < 0.05 (∗), p < 0.01 (∗∗), and p < 0.001 (∗∗∗).

## Data Availability

• The DRA accession number for WGS data reported in this paper is listed in the key resources table. • This paper does not report original code. • Any additional information required to reanalyze the data reported in this paper is available from the lead contact upon request.

## Supplemental information

Document S1. Figures S1–S4

Document S2. Article plus supplemental information
