# Supplementary material for: Generation of a tyrosine hydroxylase-2A-Cre knockin non-human primate model by homology-directed-repair-biased CRISPR genome editing
Source: Cell Rep Methods. 2023 Sep 14;3(9):100590. doi: 10.1016/j.crmeth.2023.100590 (PMC10545943; doi:10.1016/j.crmeth.2023.100590)
Supplement: Document S1. Figures S1–S4 [file mmc1.pdf]

Cell Reports Methods, Volume *3*

Supplemental information

Generation of a tyrosine hydroxylase-2A-Cre knockin non-human primate model by homology-directed-repair-biased CRISPR genome editing

Sho Yoshimatsu, Junko Okahara, Junko Yoshie, Yoko Igarashi, Ryusuke Nakajima, Tsukasa Sanosaka, Emi Qian, Tsukika Sato, Hiroya Kobayashi, Satoru Morimoto, Noriyuki Kishi, Devin M. Pillis, Punam Malik, Toshiaki Noce, and Hideyuki Okano

Supplemental information

Supplemental Figures and Legends

Figure S1. WGS analysis of TH-1 compared to the NCBI reference, related to Figure 1.

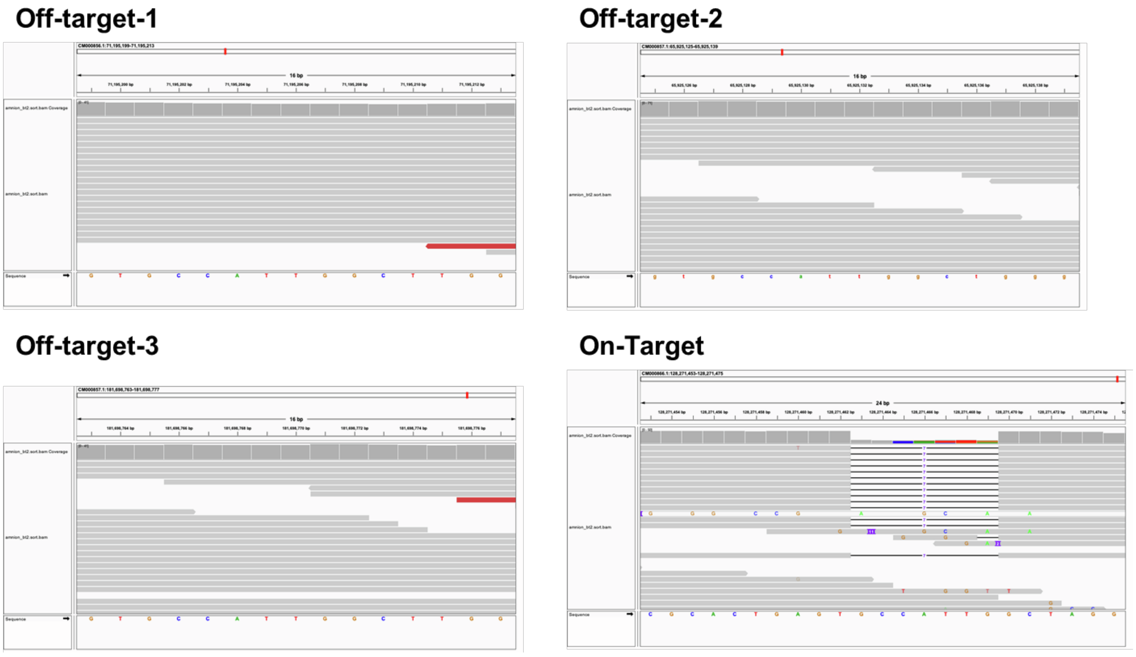

Off-target analysis of TH-1 gDNA by WGS. Off-targets were searched using CRISPRdirect[1] (http://crispr.dbcls.jp) for the TH sgRNA-1 sequence. Twenty-three potential off-target candidate regions (matching 12 nt + PAM) were found in the marmoset genomic reference (WUGSC 3.2/calJac3). Representative top-three off-target candidates and mapped reads in the regions (chr1:71195199-71195213, chr2:65925125-65925139, and chr2:181698763-181698777) were shown above. No off-targets were found among the twenty-three candidate regions. The 7-bp deletion was also detected in this analysis (lower right). Images were obtained using the Integrative Genomic Viewer[2].

Figure S2. WGS analysis of TH-1 compared to his father and maternal aunt, related to Figure 3.

A TH-1 father (CM2400M)

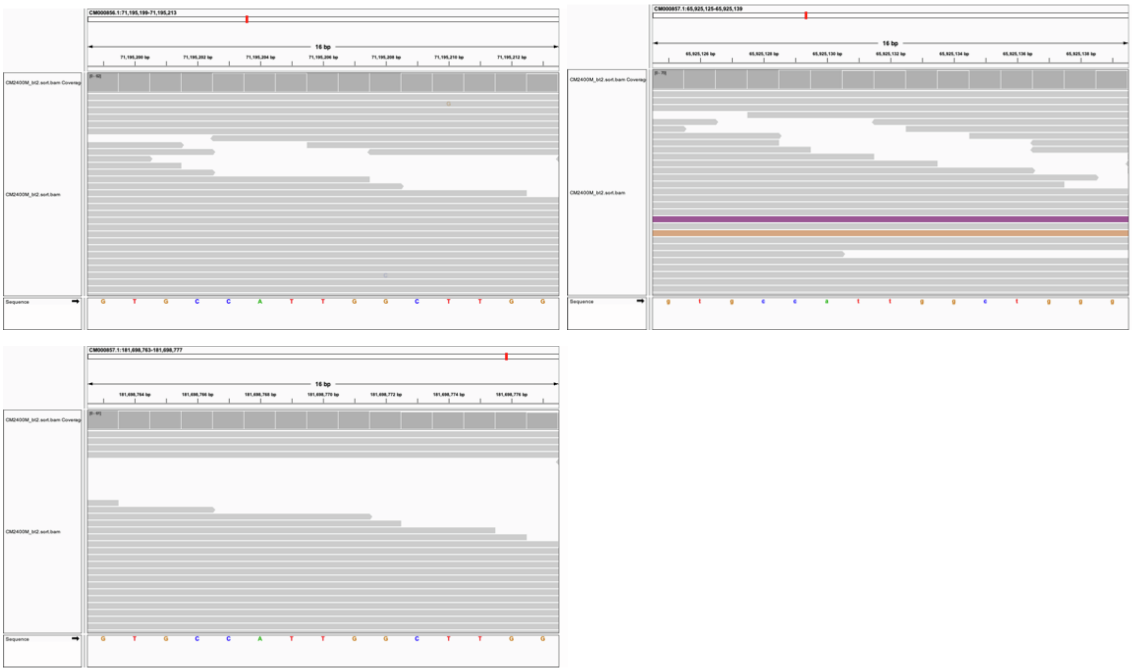

B TH-1 maternal aunt (CM1076F)

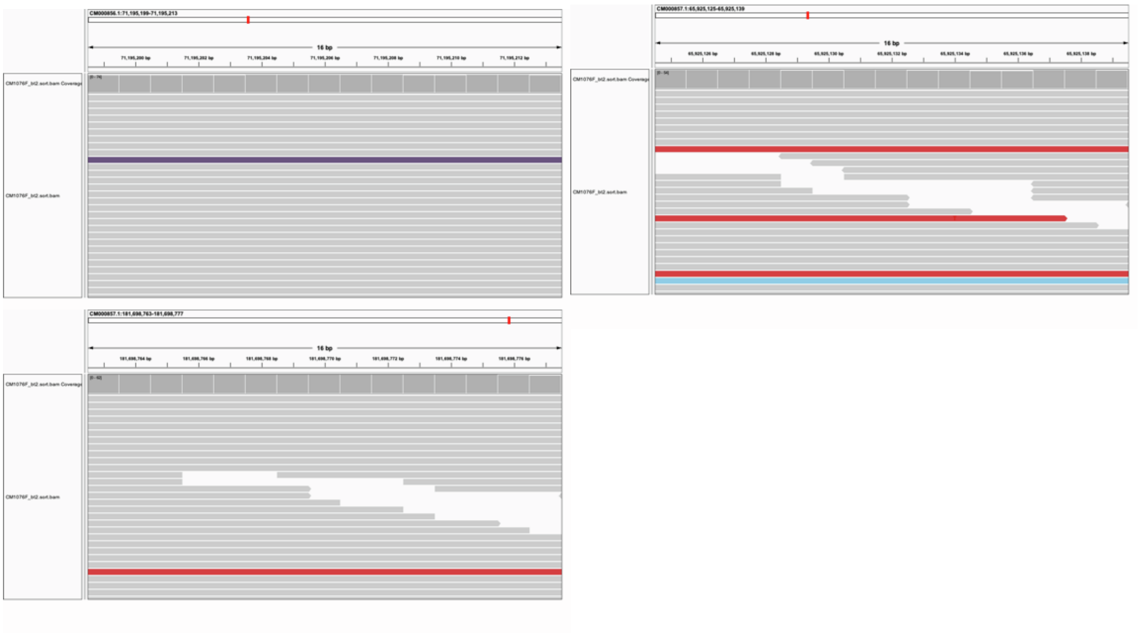

To elucidate the sequence difference between TH-1 and the reference (WUGSC 3.2/calJac3), we also performed WGS using gDNA derived from the TH-1 father (**a**; CM2400M) and maternal aunt (**b**; CM1076F) and aligned to the reference. We confirmed that there were no differences between these two animals and the reference in

the candidate off-target regions. Representative top-three off-target candidates are shown above.

Figure S3. Summary of TH-1 L-DOPA administration experiments, related to Figure 3.

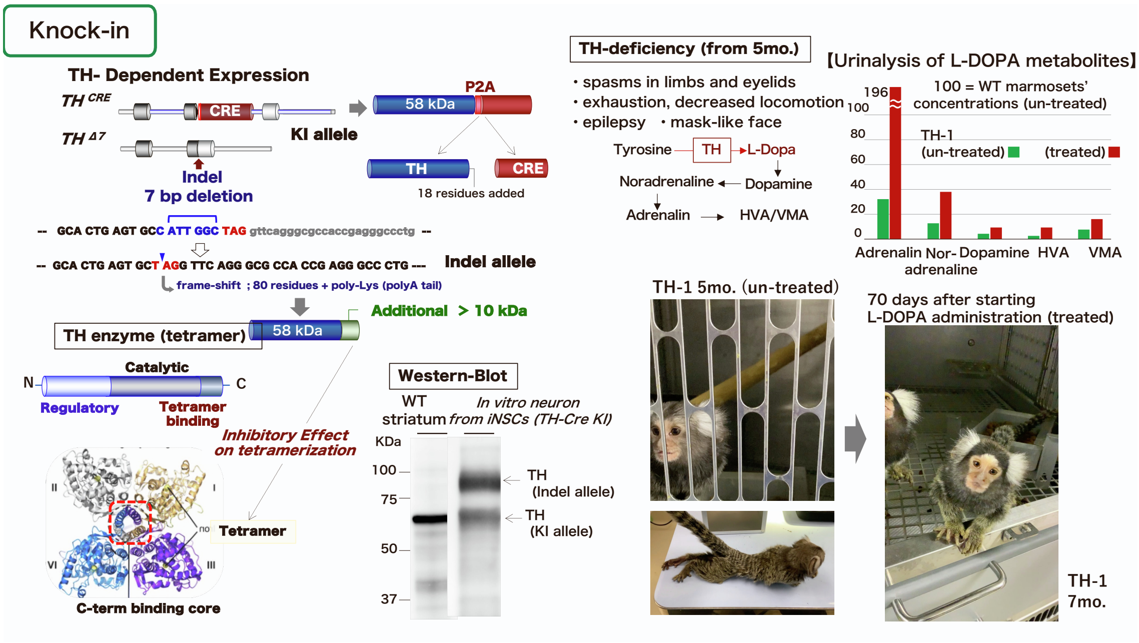

TH-1 showed a TH-deficiency-like phenotype, which may result from the inability of Cre with a 7-bp deletion (left) to compose a catalytic tetramer for the enzyme function. Therefore, we performed L-DOPA administration and found improved locomotion and increased L-DOPA-derived metabolites in urine (right, also see Video S1-3).

Figure S4. Prime editing approach for repairing *ΔCre*, related to Figure 4.

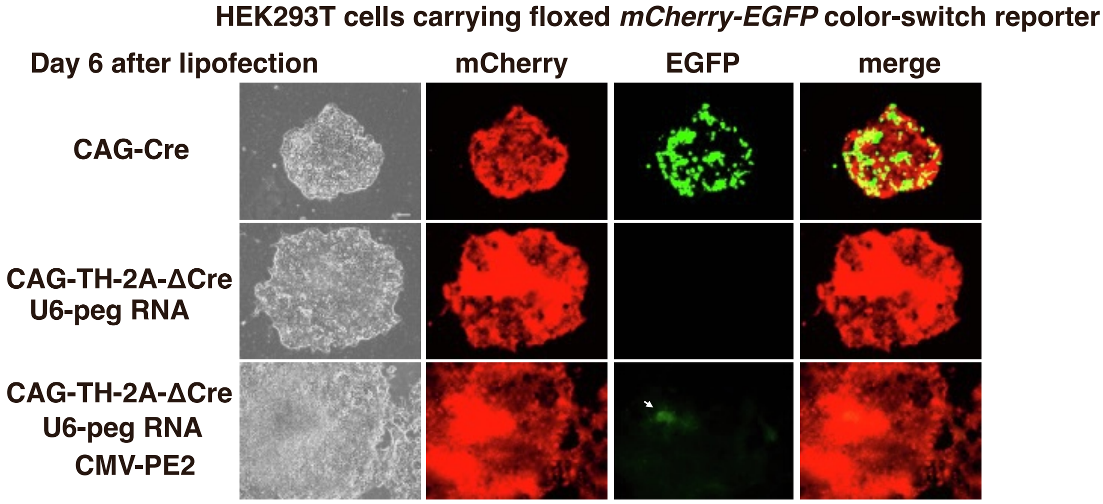

We lipofected CAG-Cre, or CAG-TH-2A-ΔCre and U6-pegRNA (Addgene #132777, specific-pegRNA subcloned) with/without CMV-PE2 (Addgene #132775) to color-switch HEK293T (used in Figure 4). The color-switch efficiency of PE2+pegRNA (bottom) was lower than 0.1%, compared to that of CAG-Cre (top). Scale bar, 100 µm. For pegRNA design, we used the PrimeDesign software[3]. For Cre-repairing pegRNA subcloning, we ligated annealed oligonucleotides to *Bsa*I-digested U6-pegRNA. Used oligonucleotides were as following: spacer-F caccGTTTCCATGAGTGAACGAACCgtttt, spacer-R ctctaaaacGGTTCGTTCACTCATGGAAAC, middle-oligo-F (5' phosphorylated) AGAGCTAGAAATAGCAAGTTAAAATAAGGCTAGTCCGTTATCAACTTGAAAAAGTGGCACCGAGTCG, middle-oligo-R (5' phosphorylated) GCACCGACTCGGTGCCACTTTTTCAAGTTGATAACGGACTAGCCTTATTTTAACTTGCTATTTCTAG, extension-F gtgcAAACAGGCTCTAGCGTTCGAACGCAcTGATTTCGAtCAGGTTCGTTCACTCATGG, and extension-R aaaaCCATGAGTGAACGAACCTGaTCGAAATCAgTGCGTTCGAACGCTAGAGCCTGTTT.

Supplemental References

1. Naito, Y., Hino, K., Bono, H., and Ui-Tei, K. (2015). CRISPRdirect: software for designing CRISPR/Cas guide RNA with reduced off-target sites. Bioinformatics *31*, 1120-1123. 10.1093/bioinformatics/btu743.
2. Robinson, J.T., Thorvaldsdottir, H., Winckler, W., Guttman, M., Lander, E.S., Getz, G., and Mesirov, J.P. (2011). Integrative genomics viewer. Nat Biotechnol *29*, 24-26. 10.1038/nbt.1754.
3. Hsu, J.Y., Grunewald, J., Szalay, R., Shih, J., Anzalone, A.V., Lam, K.C., Shen, M.W., Petri, K., Liu, D.R., Joung, J.K., and Pinello, L. (2021). PrimeDesign software for rapid and simplified design of prime editing guide RNAs. Nat Commun *12*, 1034. 10.1038/s41467-021-21337-7.
